# Potentialities of Rapid Analytical Strategies for the Identification of the Botanical Species of Several “*Specialty*” or “*Gourmet*” Oils

**DOI:** 10.3390/foods10010183

**Published:** 2021-01-18

**Authors:** Federica Turrini, Paola Zunin, Raffaella Boggia

**Affiliations:** Department of Pharmacy, University of Genoa, Viale Cembrano 4, 16148 Genoa, Italy; zunin@difar.unige.it (P.Z.); boggia@difar.unige.it (R.B.)

**Keywords:** specialty oils, fatty acids, untargeted spectroscopic fingerprint, principal component analysis, CIELab parameters, data fusion

## Abstract

A comprehensive data collection of authentic “specialty” or “gourmet” oils, namely cold-pressed industrial virgin oils, was performed. Eight different botanical species, i.e., Almond, Apricot, Avocado, Hazelnut, Mosqueta rose, Rosehip, Sunflower, and Walnut oils were studied plus Olive oil as the gold standard of cold-pressed virgin oils. Two different analytical approaches are proposed to rapidly verify the botanical species of the oil-based raw material. The first approach is based on a multivariate statistical analysis of conventional analytical data, namely their fatty acid composition. These data have been re-elaborated in a multivariate way by Principal Component Analysis (PCA) and classification methods. The second approach proposes a fast and non-destructive spectrophotometric analysis to determine the color of these oils to discriminate among different species. In this regard, the raw diffuse reflectance spectra (380–780 nm) obtained by a UV-Vis spectrophotometer with an integrating sphere was considered and elaborated by chemometrics. This information was compared with the results obtained by the most common approach based on the CIELab parameters. A data fusion of chromatographic and spectral data was also investigated. Either fatty acid composition or color of these oils demonstrated to be two promising markers of their botanical authenticity.

## 1. Introduction

Vegetable oils represent the major source of dietary fats, and oil crops are among the most significant activities of worldwide agriculture [1].

In the last decade, the so-called “specialty oils” or “gourmet oils” obtained from seeds, fruit, and nuts have had a great diffusion thanks to the increase in consumer preferences for the pleasant sensory characteristics of oils of different botanical origin. Specialty oils retain their distinctive aroma and taste because they are produced by gentle processing (gentle extraction or cold pressing) and they are not furtherly processed by conventional refining, bleaching, and deodorizing, which are routinely used to remove off-flavors and impurities as well as extend the shelf life of vegetable oils [2]. Compared to refined oils which are colorless, these virgin oils keep their original color, and sometimes, they show very intense colors (i.e., palm oil: red; corn germ oil: orange; walnut and sunflower oil: different shades of yellow) [2]. In addition to their peculiar flavor, which make them gourmet ingredients, the specialty oils contain high amounts of nutritional and functional components (such as essential fatty acids, antioxidants, phenolic compounds, phospholipids, phytosterols, etc.) responsible for their health benefits and nutraceutical properties, which make them popular in the health-promoting foods market (i.e., dietary supplements) [3,4]. Moreover, these virgin oils stand out from their refined correspondents, which are often widely distributed on the market (such as refined sunflower oils), due to their very low production from higher quality raw materials that make them quite expensive niche products.

Several studies dealing with the chemical composition, especially their fatty acid content, and the health-promoting properties of specialty oils were published [4,5,6,7]. Particularly their content of linoleic and α-linolenic acid was highlighted in several papers, while in a few papers, the study of their minor constituents was also carried out [6,8]. In 2018, Cicero et al. [8] deeply investigated the chemical composition of eight different types of these oils retailed in the Brazilian market (pequi, palm, avocado, coconut, macadamia and Brazil nut, grapeseed, and canola). Olive oil is usually used for comparison purposes, since it is considered the gold standard of the cold-pressed virgin oils, and it is the most popular specialty oil with a relative very abundant annual international production [5]. Its pleasant aroma and its healthy composition in fatty acids and minor precious compounds have allowed it to obtain a leading position in consumer preferences. It is necessary to define analytical approaches capable of guaranteeing a preliminary and rapid control tool of these “niche” oils, whose production specifications are still not so detailed, as in the case of virgin olive oils. Recently, Socaciu et al., 2020 [9] pointed out the importance of controlling the botanical authenticity of these oils, and a similar problem was already mentioned by Ozen et al. in 2003 [10]. These missing rules could favor trivial frauds in this field, including adulteration, mislabeling, characterization, and misleading origin [11]. In fact, several high-value products among the gourmet oils, such as Avocado oils and Almond oils, may be adulterated with lower-value oils, such as Sunflower oils or Apricot oils respectively, to increase profit margins [12]. Kernel oils of peach and apricot have been used as adulterants or substitutes for some expensive oils particularly, Almond oil belonging to the same botanical family (*Rosaceae*) [13]. Moreover, these adulterant oils may include some nut-containing oils, which may induce a dangerous allergic reaction for some consumers [14,15]. Furthermore, some of these specialty oils may be also sold as dietary supplement oils (DSO) and marketed for their healthy/functional properties [9,10].

The aim of this study was evaluating the possibility of identifying the botanical origin of some gourmet oils obtained from several botanical species by the routine analysis of their fatty acid composition coupled with chemometric methods and to explore also a new untargeted, fast, and non-destructive spectrophotometric analysis strategy to determine the color of the unrefined oils as a marker of their botanical authenticity. The first strategy is based on a multivariate statistical analysis of the conventional fatty acids composition. According to the degree of saturation of these carbon chains, FAs are classified as saturated (SFAs, with the maximal number of hydrogen atoms), monounsaturated (MUFAs, one double bond), and polyunsaturated (PUFAs, two or more double bonds) [16]. Principal Component Analysis (PCA) as unsupervised pattern recognition [17,18], Linear Discriminant Analysis (LDA) and *K*-Nearest Neighbors (*K*-NN) [19], as classification tools, were applied to the dataset. In the second approach, both the raw diffuse reflectance spectra recorded by a UV-Visible spectrophotometer with an integrating sphere and the results obtained by the most common colorimetric approach based on the CIELab parameters have been investigated and elaborated by chemometrics.

Finally, a data fusion combining GC-FID data with raw spectral data + SNV and the three colorimetric 260 CIELab parameters (a*, b*, L*) has been performed and discussed.

Only pure oils were considered in this preliminary study, and the complex study of the oil mixtures will be considered in a following paper.

## 2. Materials and Methods

### 2.1. Specialty Oils

Eight different specialty oils—Almond (*Prunus amygdalus* L.), Apricot (*Prunus armeniaca* L.) kernels, Avocado (*Persea americana* Mill) pulps, Hazelnuts (*Corylus avellana* L.), Mosqueta rose (*Rosa moschata*), Rosehip (*Rose hip*), Sunflower (*Helianthus annuus* L.) and Walnut (*Juglans regia* L.)—have been analyzed. With respect to Sunflower, both high and low oleic acid samples were included (namely HO and LO, respectively). In addition, Olive oil (*Olea europaea* L.) has been studied as the gold standard of cold-pressed virgin oils. These nine categories of oils (ten categories if Sunflower HO and LO samples were considered separately) were produced and marketed by an Italian Company (Parodi Nutra srl, Genoa, Italy). In detail, 226 analytical samples (see Table 1), each representative of batches of homogeneous production for raw material (e.g., with or without preliminary roasting process), agriculture (e.g., conventional or organic), and pressing conditions over a period of three years of harvest (2017–2019), have been provided.

The oil quality evaluation has been preliminary checked by several analytical parameters that are able to guarantee their commercialization as “cold press oils” and, where it applied, their consistency with the limits of Codex Alimentarius, and its proposed amendments, has been checked [20].

### 2.2. Chemicals

Analytical grade reagents were used for sample preparation and analysis. All chemicals were purchased from VWR Chemicals (Milan, Italy) and by Sigma-Aldrich (Milan, Italy).

### 2.3. Preparation and GC-FID Determination of Fatty Acid Methyl Esters (FAMEs)

FAMEs were obtained by methanolysis by KOH 2N [21]. The extracted lipids were dissolved in n-heptane to obtain a solution containing approximately 50 mg/mL lipid. First, 1 mL of this solution was transferred into a 10 mL round-bottomed tube, and then, 100 μL of 2N methanolic KOH were added. The emulsion was mixed by vortex for 1 min and then centrifuged immediately before injection. A GC-FID Agilent 7890 A has been employed for the analysis of FAMEs. The FAMEs profile was determined by split injection (70:1) on a fused silica Supelcowax 10 capillary column (30 m × 0.25 mm i.d. × 0.20 μm film thickness). The gradient temperature program started from 60 °C, which was held for 2 min; then, it was raised, at a 6 °C min^−1^ rate, up to 220 °C, and held for 20 min. Helium was the carrier gas, at a constant flow of 1.2 mL min^−1^. The injector and the detector were set at 280 °C and 300 °C respectively, with a make-up flow (He) of 25 mL min^−1^. Three injections for each extract were performed.

### 2.4. Spectroscopic Analysis

An UV-Visible spectrophotometer Cary 100 (Varian Co., Palo Alto, CA, USA) equipped with a Varian DRA integrating sphere and with a solid sample holder was employed. UV-Visible spectra, in the 380–780 nm range at a resolution of 1 nm, were collected using a white Spectralon^®^ disk as reference. Quartz cuvettes SUPRASIL^®^ 300 (Hellma Mullheim, Germany) with rectangular-section cells, 1 cm path length and 3.5 mL volume capacity have been used. Samples were acquired randomly, and three replicates of the diffuse reflectance for each sample were recorded and averaged to minimize unwanted spectral variability. The CIELab coordinates: L* (lightness), a* (reddish–greenish), and b* (yellowish–bluish) of all sample analyzed were automatically calculated from the raw spectral data by the Cary 100 color software using the CIE D65 illuminant. 

### 2.5. Multivariate Statistical Analysis

Multivariate statistical Analysis was performed by the *Chemometric Agil Tool* (CAT), an R-based chemometric software developed by the Chemistry Group of the Italian Chemical Society [22] and by *PLS*-Toolbox (Eigenvector, https://eigenvector.com/software/pls-toolbox/). Principal Component Analysis (PCA) has been applied as common multivariate statistical method of unsupervised pattern recognition to simplify and visualize data by extracting only the important information from the dataset [23,24]. Standard normal variate (SNV) transform coupled to column centering have been previously performed on the spectral data to remove multiplicative effects of scattering and to scale the data, respectively [25]. The ten categories were classified all together using two classification techniques (LDA and KNN) aiming to discriminate among many classes all together, whose numerosity is quite heterogeneous. The classification results have been tested both internally by means of internal five-fold cross-validation with venetian blinds splitting of the training samples and by an external test set (external prediction). Linear Discriminant Analysis (LDA) was applied as a probabilistic classification technique that searches for directions (canonical variables) with maximum separation among multiple categories (the nine different botanical species) [19]. The *K*-Nearest Neighbors (*K*-NN) algorithm was applied as a non-linear classification method based on distances among samples. It predicts the class membership of a test sample based on the class of the k nearest sample(s) in the multidimensional space [26]. In the present study, the number of *k* neighbors (*k* = 5) was chosen according to the best classification rate in an optimization performed by a cross-validation scheme.

## 3. Results and Discussion

### 3.1. FAMEs Composition of Specialty Oils

This research concerns the analysis of some gourmet oils belonging to eight different botanical species (Almond, Apricot, Avocado, Hazelnut, Mosqueta rose, Rosehip, Sunflower, Walnut). These botanical species have been considered for their expanding employment as “gourmet” oils. More than two hundred “authentic” industrial samples of cold-pressed virgin oils coming from controlled supply chains were studied plus olive oils samples used for comparison purposes, since they are the “gold standard” for edible virgin oils.

The fatty acid profile of the oils analyzed is shown in Table 2 and in Table S1 (Supplementary Materials). Sunflower oils, both low and high oleic, are characterized, compared to all the others, by higher values of behenic (C22:0) and lignoceric (C24:0) acids, which are completely absent in many other botanical species. Mosqueta rose and Rosehip oils are those with the highest value of α-linolenic acid (C18:3) and with the presence of its γ-isomer, too. Walnut oils follow rose oils in C18:3 content and are also characterized by high C18:2 value. Avocado oils are characterized by higher values of palmitoleic acid (C16:1) and palmitic acid (C16:0) compared to Almond, Apricot, Olive, and the other oils. Hazelnut oils with respect to Apricot and Almond oils are characterized by lower oleic (C18:1) and stearic acid (C18:0) contents and higher linoleic (C18:2) contents. Olive and Hazelnut oils present the highest content in oleic acid (C18:1) compared with all the other oils, but Hazelnut oils have also higher values of C18:2 and lower values of C16:0 when compared to Olive samples.

Furthermore, it is important to point out that also trans-fatty acids have been checked, and that their content appeared to be completely absent in all the samples except for Walnut oils [27,28], whose low but detectable content of *trans*-fatty acids makes this category distinguishable from all the others.

**Figure 1:**
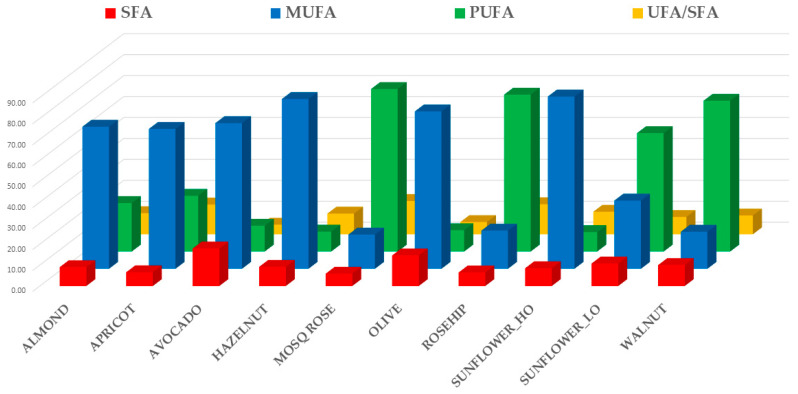
Mean fatty acid composition of different specialty oils. Saturated fatty acids (SFA), monounsaturated fatty acids (MUFA), polyunsaturated fatty acids (PUFA), and the ratio between unsaturated and saturated fatty acids (UFA/SFA) were highlighted.

Figure 1 showed the total amount of saturated fatty acids (SFAs), monounsaturated fatty acids (MUFAs), polyunsaturated fatty acids (PUFAs), and the ratio between unsaturated and saturated fatty acids (UFA/SFA) of analyzed gourmet oils.

Avocado and Olive oils have the highest amounts of SFAs, equal to approximately 18 ± 2.3% and 15 ± 1.5% of total fatty acids, respectively. On the contrary, Mosqueta, Rosehip, and Apricot oils have the lowest SFA contents (approximately < 8%). Palmitic acid (C16:0) is always predominant, followed by steric acid (C18:0). Sunflower_HO, Hazelnut, and Olive oils have the highest MUFA contents (average values approximately between 75% and 82%), followed by Avocado, Apricot and Almond oils (average values approximately between 66 and 70%). Mosqueta rose, Rosehip, and Walnut have the lowest MUFAs content (<21%) and, at the same time, the highest PUFA levels (average values equal to 77.6, 74.9, and 72.0%, respectively). Oleic acid (C18:1) is always predominant among MUFAs, while the content of other MUFA acids is very low (approximately < 1%) except for palmitoleic acid (C16:1) in Avocado oils, whose average value reaches about 6%. As for PUFAs, linoleic acid (C18:1) is always predominant over linolenic acid (C18:2), although Rosehip oils have closer levels of these two acids (44.1 and 32.7% respectively as average values). Concerning the UFA/SFA ratio, the lowest levels have been detected in Avocado and Olive oils (average values approximately < 6%), while the highest ones are typical of Mosqueta, Rosehip, and Apricot oils (average values approximately > 14%). This high ratio is often exploited for the vaunted health properties of these niche products, which are often used as dietary supplements as well as gourmet oils [29].

### 3.2. PCA on Fatty Acid Composition of Specialty Oils

As far as fatty acid composition is concerned, two data matrixes were employed: the former, named A_204,18_, involves 204 rows (the oil samples employed to build the models) and 18 columns (the detected FAMEs) (Table 2), the latter B_27,18_ involves 27 rows (the oil samples employed to test the models) and the 18 FAMEs.

**Figure 2:**
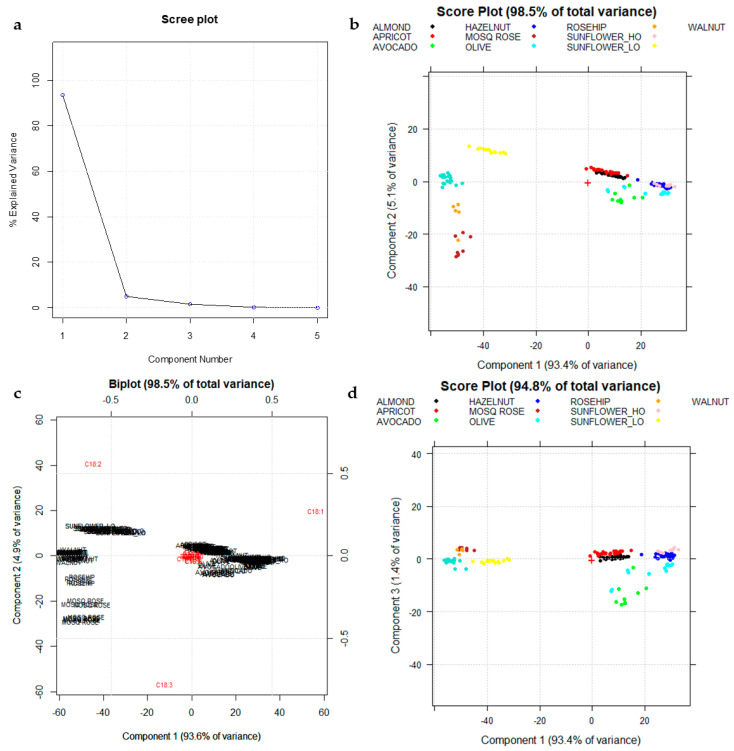
(**a**) Scree plot of A_204,18_; (**b**) Score plot on the first two principal components (PC1–PC2) calculated using the training set (204 samples). Different species are highlighted in different colors. (**c**) Biplot (score and loading plot) on PC1–PC2; (**d**) Score plot on PC1–PC3. Different species are highlighted in different colors.

Principal Component Analysis (PCA) was applied as an exploratory tool of the data structure, and it was performed on a training set of 204 oil samples, and the other 22 samples have been used as test set for an external prediction. The Scree plot (Figure 2a) highlights as the first three principal components (PCs) retain more than 99% of the total variance using mean-centered data. PCA diagnostic (Scree plots and influence plots) has been reported in the Supplementary Materials (Figure S1).

Figure 2b shows the score plot on the first two principal components (PC1 vs. PC2) calculated using the training set (204 samples) after column centering as the preprocessing method. Column centering is allowed, since all variables have the same unit of measure. As highlighted in Figure 2b,c, two main groups of oils are highlighted: the low oleic (C18:1) group with lower scores on PC1 (Sunflower_LO, Walnut, Rosehip, and Mosqueta Rose) and the high oleic (C18:1) group with higher scores on PC1 (Olive, Avocado, Sunflower_HO, Apricot, Almond, and Hazelnut). The low oleic (C18:1) and high linoleic (C18:2) groups separate from each other on PC2 depending on their linolenic acid (C18:3) content (Figure 2c). Mosqueta rose and Rosehip oils are those with the highest value of α-linolenic acid (C18: 3) and with the presence of its γ-isomer, too (Table 2). Moreover, Walnut oils follow rose oils in C18:3 content. 

The PC1 vs. PC2 plot (Figure 2b,c) highlights the separation among the different low oleic categories, while the PC1 vs. PC3 plot (Figure 2d) highlights the separation of Avocado oils (in green ink) from the remaining high-oleic categories (with high scores on PC1).

**Figure 3:**
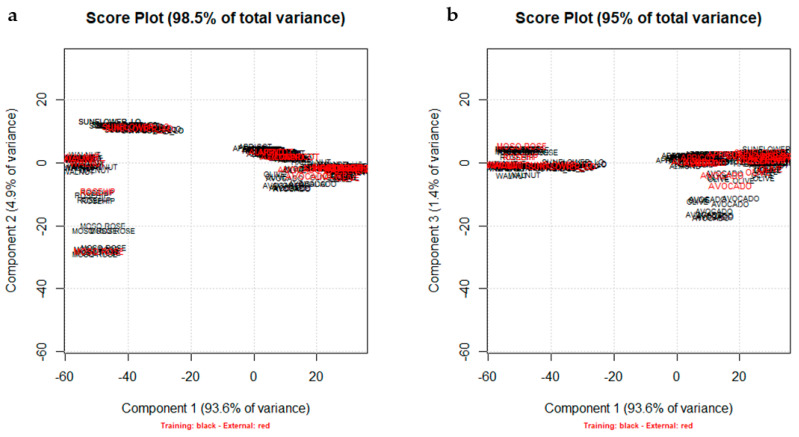
The projection of the external test set (B_27,18_: red ink) on PC1–PC2 (**a**) and PC1–PC3 (**b**) score plots, respectively.

Figure 3a,b shows the projection of the external test set (22 samples) on PC1–PC2 and PC1–PC3 score plots, respectively, confirming a good separation of the low oleic (C18:1) botanical species (Sunflower_LO, Walnut, Rosehip, and Mosqueta Rose) and of the Avocado group, due to their different pattern of majority fatty acids as highlighted by the PC1–PC2 and PC1–PC3 score plots (Figure 2b,d).

**Figure 4:**
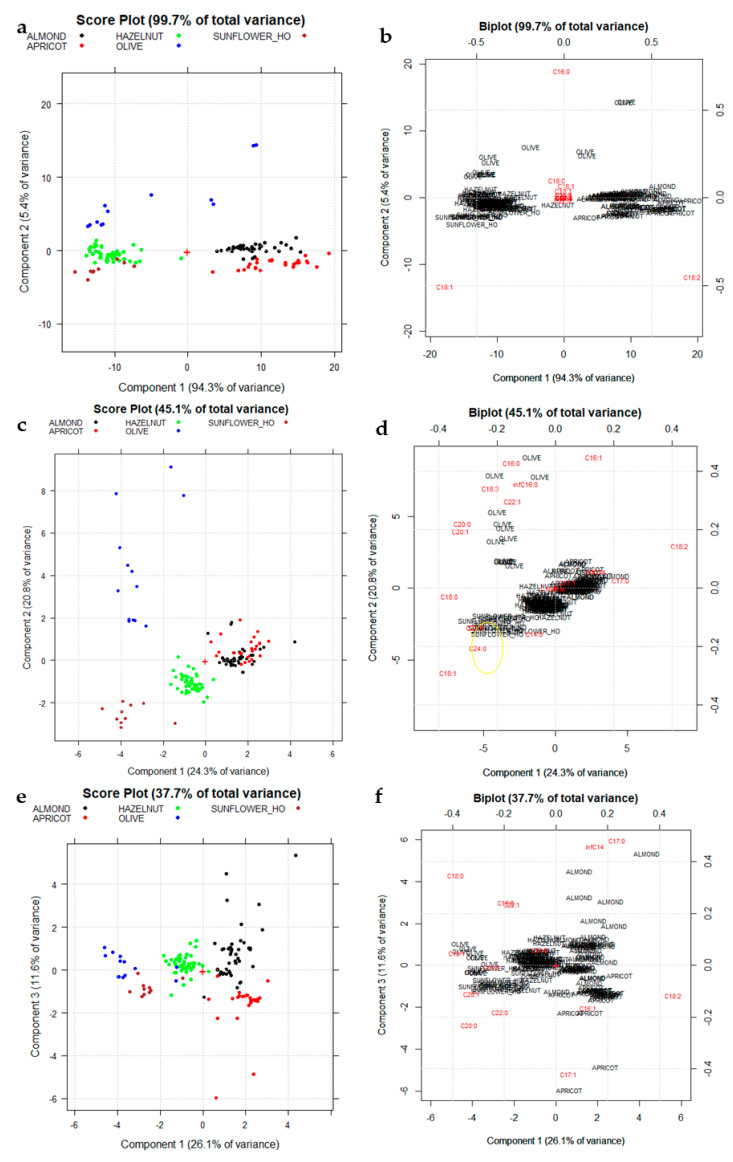
(**a**) Score plot on the first two principal components (PC1 vs. PC2) data matrix C_140,18_ after column centering. Each vegetable species is reported with a different color (Almond: black, Apricot: red, Hazelnut: green, Olive: blue, Sunflower_HO: brown); (**b**) Biplot on PC1–PC2 after column-centering: loadings are reported in red ink; (**c**) Score plot on the first two principal components (PC1 vs. PC2) data matrix C_140,18_ after autoscaling; (**d**) Biplot on PC1–PC2 after autoscaling: loadings are reported in red ink; (**e**) Score plot on PC1–PC3 data matrix C_140,18_ after autoscaling; (**f**) Biplot on PC1–PC3 after autoscaling: loadings are reported in red ink. + sign in the plots correspond to the origin (point 0,0).

Then, the five unresolved high oleic (C18:1) categories, such as Olive, Sunflower_HO, Apricot, Almond, and Hazelnut, have been separately considered (Figure 4). Another PCA has been performed just using these five categories comparing both the column centering and the autoscaling as data pre-treatments. A new matrix named C_140,18_ involves 140 rows (the oil samples belonging to the five categories under study) and 18 columns (the detected FAMEs) has been prepared. PCA diagnostic (Scree plots and influence plots) has been reported in the Supplementary Materials (Figure S2).

Figure 4a,b shows the fatty acid profiles, after column centering, recorded for the five vegetable species investigated: Almond (black ink), Apricot (red ink), Hazelnut (green ink), Olive (blue ink), and Sunflower_HO (brown ink). The first two PCs of the dataset (T_140,18_), which together explained 99.7% of the total information of the dataset since they visualize almost the 100% of the total variance, have been considered. The PC1–PC2 score plot (Figure 4a,b) highlights a moderate separation among the five categories of gourmet oil investigated. Particularly PC1, the direction of maximum variance which explains almost the 95% of the total information, allows a discrimination between the relatively higher oleic (C18:1) classes (Sunflower_HO and Hazelnut) and the relatively higher linoleic (C18:2) classes such as Almond and Apricot. Furthermore PC2, which explains the 5.4% of the remaining variance, allows slightly separating Hazelnut (green ink) from Sunflower_HO (brown ink) and Almond oils (black ink) from Apricot ones (red ink), respectively due to different C16:0 contents. 

Figure 4c,d shows the PC1-PC2 score plot, which explain the 45.1% of the total variance, obtained from the above-mentioned data matrix after autoscaling, which allows highlighting the contribution of minor fatty acids. In Figure 4c, all the considered botanical species are separated with the exception of Apricot oils (red ink) and Almond oils (black ink), which remain confused. Figure 4d shows that Sunflower oils, both low and high oleic, are characterized, compared to all the others, by higher values of behenic (C22:0) and lignoceric (C24:0) acids (highlighted in yellow), which are completely absent in many other botanical species (see Table 2).

Figure 4e,f shows the PC1–PC3 score plot, which explain the 37.7% of the total variance obtained from the above-mentioned data matrix after autoscaling. A discrimination between Almond and Apricot has been obtained mainly due to a slightly higher content in C16:0 and C18:0 of Almond with respect to Apricot oils.

The samples having both high Q and T^2^ values in the influence plot correspond to samples (two olive sample, namely BIO_C_0028C16 and BIO_C_0028B17, in the first dataset A_204,18_; one apricot sample, BIO_C_0033F17 in the second dataset C_140,18_) having C18:1 and C18:2 to the limit values of the range of these variables, which are largely influenced by different geographical origin (i.e., Olive oils) [30] and by maturity index (i.e., Apricot) [31].

Then, a focus on these last more critical two species have been performed. A new matrix D_70,14_ which involves 70 rows (the oil samples belonging to Almond and Apricot categories) and 14 columns (corresponding to the detected FAMEs different from zero) has been prepared and analogously analyzed by PCA after column centering. The related PCA Diagnostic (Influence plots) has been reported in the Supplementary Materials (Figure S3).

**Figure 5:**
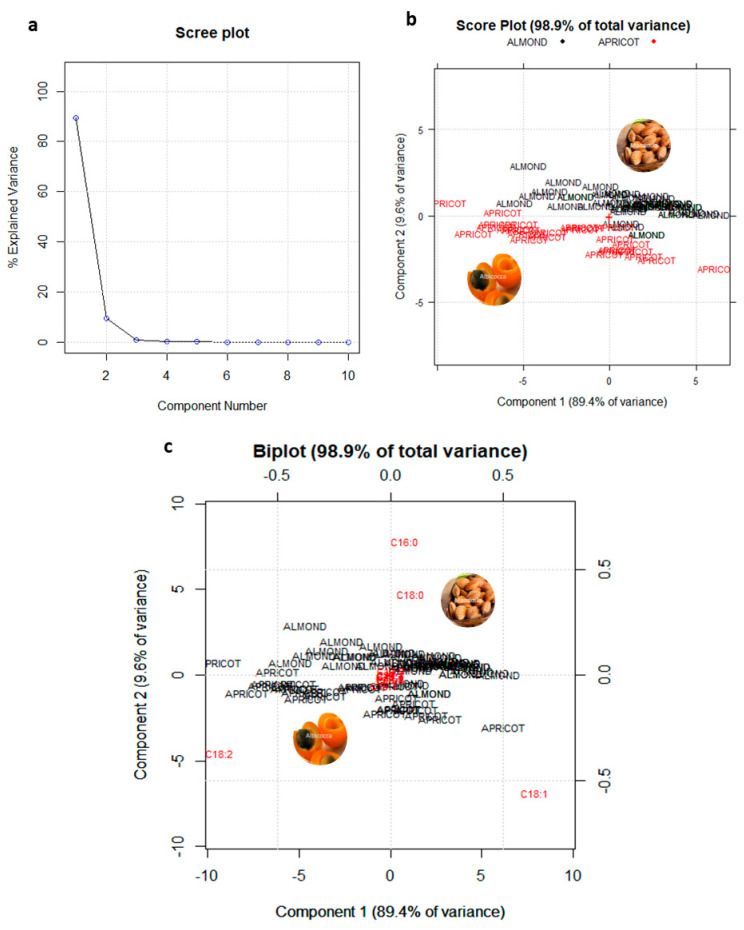
(**a**) Scree plot; (**b**) Score plot on PC–PC2; (**c**) Biplot (score and loading plot) on PC1–PC2.

Figure 5 shows the Scree plot, the Score plot PC1–PC2, and the corresponding Biplot. Figure 5a shows that the first three PCs retain more than 99% of the total variance. Figure 5b highlights a quite good separation between Almond (in black ink) and Apricot (in red ink) on PC2, which explains 9.6% of the total variance of the dataset. Looking at the Biplot (Figure 5c), it is more clearly evident than previously cited as Almond oils contain higher amounts of palmitic acid (C16:0) and stearic acid (C18:0) with respect to Apricot ones (see Table 2).

### 3.3. Classification Analysis

Linear Discriminant Analysis (LDA) and *K*-Nearest Neighbors (*K*-NN) classification results are reported in Table 3.

LDA results confirms the fatty acids composition, previously and opportunely scaled, as a marker of the botanical species of these specialty oils with a 95.4% of correct predictions in cross-validation (five cross-validation groups, internal prediction) and 100% of corrected predictions of the test set oils (external prediction).

Regarding *K*-NN classification, the best results for the *k* variables between 1 and 7 are obtained with *k* = 5. All botanical species, except for Rosehip, are well predicted in internal validation (five cross-validation groups, 84.7% of correct predictions in cross-validation). Instead, in external prediction, Avocado, Olive, and Rosehip oils remain confused with each other with an 80% of corrected predictions of the test set oils.

In Table 4, the classification analysis performed only on the two more “critical” classes (Almond and Apricot) was reported. Further details of the classification results (i.e., sensitivity, specificity, and confusion matrix) are reported in the Supplementary Materials (Table S2). Both LDA and *K*-NN (*k* = 5) results were satisfactory with the exception of Rosehip oils, which remain confused with the other rose oils (Mosqueta Rose).

### 3.4. Color Analysis and PCA Analysis

As far as the color analysis is concerned, only 37 samples were pressed within a few months before the analysis were considered in order to evaluate their color before its degradation due to time, which could represent a limit to the use of this analytical strategy. Rosehip samples were not available freshly pressed; therefore, this category was not studied in this second dataset.

Two data matrices were employed: the former named E_37,400_ involves 37 rows (corresponding to the oil samples analyzed) and 400 columns (the reflectance variables recorded in the spectral range 380–780 nm) and the latter F_37,3_ involves 37 rows (oil samples) and three columns corresponding to the three CIELab parameters (a*, b*, L*) obtained from the spectral data.

**Figure 6:**
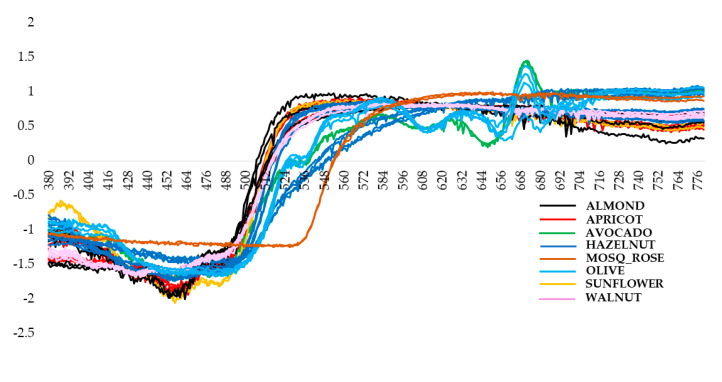
UV-Visible spectra of the different vegetal species after standard normal variate (SNV) pre-treatment of data.

The corresponding spectra (380–780 nm) are shown in Figure 6 after the SNV pretreatment.

**Figure 7:**
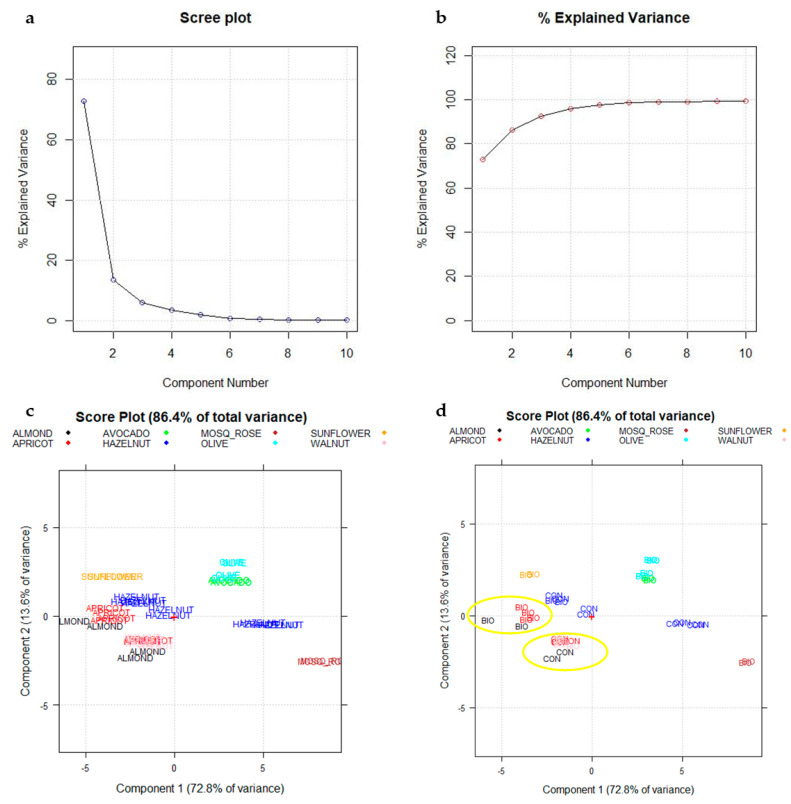
(**a**,**b**) Scree plot and percentage of explained variance plot; (**c**) Score plot PC1–PC2. Each vegetable species is reported with a different color; (**d**) Conventional (CON) and biological (BIO) samples have been highlighted. + sign in the plots correspond to the origin (point 0,0).

The PCA results on the 400 raw spectral variables (G_37,400_), after SNV and mean centering, are shown in Figure 7.

Figure 7a,b reports the Scree plot and the cumulative plot (% of explained variance plot), respectively. The first two PCs together explain more than the 86% of the total information of the dataset, since they visualize the 86.4% of the total variance. Figure 7c,d shows how oils from different botanical species are moderately separated on the first two PCs. Furthermore, for the categories Almond (in black), Apricot (in red), and Hazelnut (in blue), a further subdivision into subgroups should be noted due to the different spectral behavior of conventional (CON) samples compared to biological (BIO) samples. This is more evident for Almond and Apricot species, whose biological samples (BIO) showed lower scores on PC1 and higher scores on PC2 with respect to the corresponding conventional samples (CON) belonging to the same botanical class (Figure 7d, highlighted in yellow).

Regarding Hazelnut species, a greater number of samples (Table 1) and higher heterogeneity in these ones have been showed. In addition to the botanical species, cultivar and the geographical origin may also play a decisive role in the variability of the color of these latest oils.

This separation is not worst when, instead of using raw spectra data, the three colorimetric CIELab parameters (a*, b*, L*) have been processed in a similar way. In this case, a great confusion among the different botanical species was highlighted, and only a few botanical categories can be grouped (data reported in the Supplementary Materials).

### 3.5. PCA on FAMEs Composition Coupled to Spectroscopic Data and CIELab Parameters (Data Fusion)

Finally, PCA was performed on the fused data matrix H_37,421_, which combines the chromatographic data (FAMEs composition, 18 variables), with the raw spectral data after SNV pretreatment (400 variables), plus the three CIELAB descriptors (a*, b*, L*). Block scaling treatment (three blocks) was previously performed to scale the dataset and consider in the data analysis the same importance for all the variables [32,33].

**Figure 8:**
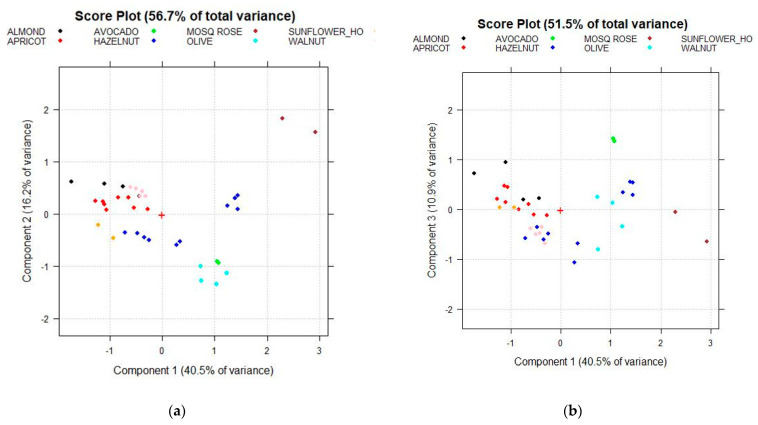
Score plots of the fatty acid methyl esters (FAMEs) composition, coupled to the raw spectral data after standard normal variate (SNV) pretreatment, and the CIELAB descriptors (data matrix H_37,421_): (**a**) PC1–PC2 Score plot; (**b**) PC1–PC3 Score plot. + sign in the plots correspond to the origin (point 0,0).

Both the PC1–PC2 score plot (Figure 8a), which explains the 56.7% of the total variance of the dataset, and the PC1–PC3 score plot (Figure 8b), which explain the 51.5% of the total variance, allow separating the Mosqueta rose (in brown ink), Avocado (in green ink), and Olive (in light blue ink) classes. As concerns Hazelnut oils, a subdivision into two subgroups was highlighted, as previously showed in Figure 7c,d.

**Figure 9:**
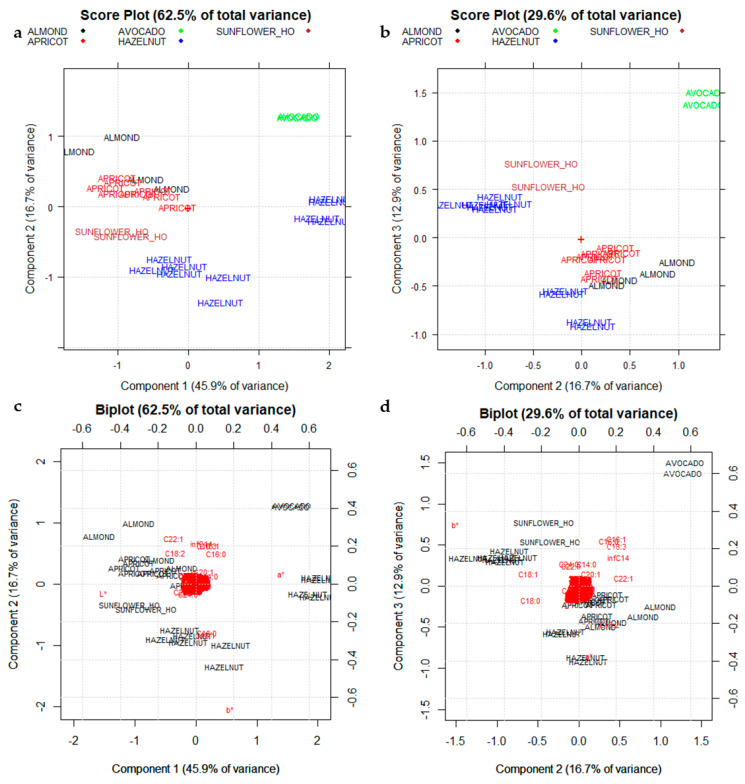
Score plots of the FAMEs composition, coupled to the raw spectral data after SNV pretreatment, and the CIELAB descriptors (data matrix I_28,418_): (**a**) PC1–PC2 Score plot; (**b**) PC2–PC3 Score plot; (**c**) PC1–PC2 Biplot; (**d**) PC2–PC3 Biplot. + sign in the plots correspond to the origin (point 0,0). The three CIELab parameters: L* (lightness), a* (reddish–greenish), and b* (yellowish–bluish).

The five unresolved classes, such as Almond, Apricot, Hazelnut, Sunflower_HO, and Walnut have been separately considered (Figure 9). A new matrix named I_28,418_ consisting of 28 rows (the oil samples belonging to these five categories) and 414 columns (corresponding to the 15 detected FAMEs coupled to the 400 spectral variables, plus the three CIELAB parameters) has been prepared and analogously analyzed by PCA.

Figure 9a,b highlights a separation among the five vegetal species considered, except for Hazelnut oils which, as previously discussed, are divided into two different subgroups. As highlighted in the corresponding Biplot (Figure 9c,d), the two different subgroups of Hazelnut oils show different color (different parameters of a* and b*). 

This approach that involves PCA on chromatographic data (FAMEs composition) coupled to spectroscopic data (reflectance spectra) and to the CIELab parameters (a*, b*, L) allows solving the sample discrimination of specialty oils and could be a promising strategy to evaluate their botanical authenticity. PCA diagnostic (Scree plots and influence plots) has been reported in the Supplementary Materials (Figure S4).

## 4. Conclusions

Specialty or gourmet oils are increasingly appreciated in the oil scenery. Raw materials such as seeds, fruit, and nuts are interesting and valuable sources to produce gourmet oils usable, as suppliers of essential fatty acids and other bioactive compounds, in human nutrition or technical applications.

The lack of detailed production specifications for these “niche” oils makes these products at high risk of fraud and adulteration. For this reason, there is an increase demand to identify analytical strategies to characterize the botanical origin of these oils. These strategies aim not only to safeguard the market from illegal practices but also to provide an additional tool in the industrial production chain.

The fatty acid composition represents a conventional and low-cost analysis that could be used to discriminate among different botanical species of specialty oils if processed using a multivariate approach (PCA and classification methods). This same statistical multivariate approach could be also applied to the raw spectral data coming from the color analysis of the same oils, if freshly pressed. Processing the raw spectral data rather than the CIELab coordinates automatically obtained from them, has shown promising results for the rapid and non-destructive identification of the botanical species of these gourmet oils. Moreover, also, chromatographic data (fatty acid composition) coupled to spectroscopic data (reflectance spectra) and to the CIELab parameters (a*, b*, L), if analyzed by PCA allow solving the sample discrimination of specialty oils representing a promising strategy to check their botanical authenticity. In addition to the botanical species, other factors such as cultivar and geographical origin can also play a decisive role in the variability of the color of these oils, offering further research ideas in this area.

## Figures and Tables

**Table 1:** Sample information: number of samples, type of farming, and preliminary roasting treatment undergone for specialty oils belonging to each botanical species studied.

SPECIALTY OIL	Number of Samples	Toasting Treatment	Farming	
			Organic	Conventional
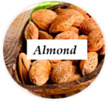	48		34	14
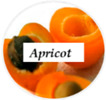	27		9	18
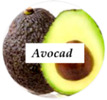	11		5	6
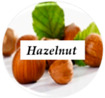	51	18	8	43
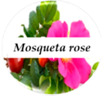	9		6	3
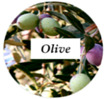	15		15	-
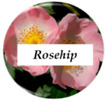	6		4	2
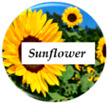	33 HO ^a^: 12 LO ^b^: 21		29	4
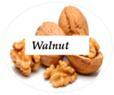	26	2	13	13

^a^ High oleic; ^b^ Low oleic.

**Table 2:** Individual fatty acid composition, total saturated fatty acids (SFA), monounsaturated fatty acids (MUFA), polyunsaturated fatty acids (PUFA) contents and unsaturated and saturated fatty acids (UFA:SFA) ratio of analyzed gourmet oils. Results are reported as peak area percent ± uncertainty expressed as the half-width of the 95% confidence interval.

	*Almond*	*Apricot*	*Avocado*	*Hazelnut*	*Mosq Rose*	*Olive*	*Rosehip*	*Sunflower_HO*	*Sunflower_LO*	*Walnut*
infC14	0.01 ± 0.01	nd	nd	nd	nd	nd	nd	nd	nd	nd
C14:0	0.01 ± 0.01	nd	0.04 ± 0.03	0.01 ± 0.01	nd	nd	0.02 ± 0.03	0.02 ± 0.02	0.05 ± 0.02	0.03 ± 0.00
infC16:0	nd	nd	nd	nd	nd	0.02 ± 0.02	nd	nd	nd	nd
C16:0	6.57 ± 0.10	5.06 ± 0.17	16.89 ± 2.52	6.39 ± 0.16	3.64 ± 0.12	11.08 ± 1.70	3.77 ± 0.57	4.39 ± 0.28	6.49 ± 0.19	7.20 ± 0.41
C16:1	0.50 ± 0.03	0.79 ± 0.06	6.10 ± 1.50	0.29 ± 0.02	0.11 ± 0.04	0.92 ± 0.33	0.10 ± 0.13	0.15 ± 0.02	0.15 ± 0.04	0.11 ± 0.02
C17:0	0.05 ± 0.01	0.01 ± 0.01	nd	0.01 ± 0.01	nd	nd	nd	nd	nd	nd
C17:1	nd	0.01 ± 0.01	0.01 ± 0.01	nd	nd	nd	nd	nd	nd	nd
C18:0	2.26 ± 0.13	1.30 ± 0.09	0.85 ± 0.22	2.63 ± 0.09	1.85 ± 0.12	3.02 ± 0.20	2.25 ± 0.35	2.86 ± 0.34	3.15 ± 0.16	2.61 ± 0.10
C18:1	67.23 ± 0.59	65.88 ± 1.18	63.32 ± 3.25	80.65 ± 0.46	15.96 ± 1.54	73.88 ± 4.69	17.86 ± 1.76	81.86 ± 1.59	32.25 ± 1.42	17.47 ± 0.62
C18:2	23.04 ± 0.59	26.47 ± 1.10	11.47 ± 2.03	9.45 ± 0.53	44.12 ± 1.32	9.58 ± 3.01	51.42 ± 4.08	9.13 ± 1.11	56.41 ± 1.41	60.25 ± 0.92
C18:3	0.10 ± 0.02	0.14 ± 0.04	0.84 ± 0.14	0.07 ± 0.03	32.71 ± 2.91	0.61 ± 0.14	23.03 ± 5.47	0.16 ± 0.05	0.14 ± 0.03	11.72 ± 0.55
C18:3 is.	nd	nd	nd	nd	0.77 ± 0.38	nd	0.43 ± 0.32	nd	nd	nd
C20:0	0.09 ± 0.01	0.17 ± 0.05	0.10 ± 0.03	0.16	0.19 ± 0.27	0.41 ± 0.06	0.23 ± 0.35	0.24 ± 0.07	0.23 ± 0.01	0.14 ± 0.06
C20:1	0.09 ± 0.01	0.11 ± 0.02	0.12 ± 0.07	0.11	0.20 ± 0.12	0.30 ± 0.05	0.24 ± 0.18	0.23 ± 0.07	0.18 ± 0.04	0.12 ± 0.04
C22:0	0.03 ± 0.01	0.06 ± 0.04	0.05 ± 0.04	nd	0.18 ± 0.06	0.11 ± 0.02	0.17 ± 0.06	0.77 ± 0.08	0.64 ± 0.05	0.04 ± 0.03
C22:1	0.01 ± 0.01	nd	nd	nd	0.08 ± 0.03	0.04 ± 0.03	0.03 ± 0.04	nd	nd	nd
C24:0	nd	nd	nd	nd	0.05 ± 0.04	nd	0.05 ± 0.10	0.24 ± 0.06	0.23 ± 0.03	nd
∑ *trans*	nd	nd	nd	nd	nd	nd	nd	nd	nd	0.30 ± 0.19
SFAs	9.02 ± 0.20	6.60 ± 0.21	17.93 ± 2.33	9.19 ± 0.22	5.91 ± 0.31	14.63 ± 1.56	6.50 ± 0.62	8.52 ± 0.66	10.79 ± 0.31	10.02 ± 0.40
MUFAs	67.70 ± 0.59	66.79 ± 1.14	69.55 ± 2.23	81.05 ± 0.45	16.35 ± 16.28	75.14 ± 4.42	18.23 ± 1.75	82.24 ± 1.58	32.57 ± 1.44	17.71 ± 0.56
PUFAs	23.14 ± 0.59	26.61 ± 1.10	12.31 ± 2.06	9.52 ± 0.53	77.59 ± 2.08	10.19 ± 3.01	74.88 ± 2.08	9.29 ± 1.14	56.54 ± 1.41	71.96 ± 0.86
UFA/SFA	10.08	14.16	4.57	9.86	15.89	5.83	14.32	10.74	8.26	8.95

nd: not detectable.

**Table 3:** Linear Discriminant Analysis (LDA) and *K*-Nearest Neighbors (*K*-NN, *k* = 5) classification results.

	LDA		*K*-NN	
Specialty Oil	% Correct Predictions in Cross-Validation (CV)	% Correct Predictions	% Correct Predictions in Cross-Validation (CV)	% Correct Predictions
*Almond*	91.1	100	91.1	100
*Apricot*	96	100	88.0	100
*Avocado*	88.9	100	88.9	50
*Hazelnut*	97.9	100	91.5	100
*Mosq Rose*	100	100	87.5	100
*Olive*	100	100	92.3	50
*Rosehip*	80	100	20.0	0
*Sunflower_HO*	100	100	90.0	100
*Sunflower_LO*	100	100	100.0	100
*Walnut*	100	100	100.0	100
	% Total Correct Predictions in CV	% Total Correct Predictions	% Total Correct Predictions in CV	% Total Correct Predictions
	95.4	100	84.7	80

**Table 4:** Linear Discriminant Analysis (LDA) and *K*-Nearest Neighbors (*K*-NN, *k* = 5) classification results on Almond and Apricot classes.

	LDA		*K*-NN	
Specialty Oil	% Correct Predictions in Cross-Validation (CV)	% Correct Predictions	% Correct Predictions in Cross-Validation (CV)	% Correct Predictions
*Almond*	100	100	97.73	97.77
*Apricot*	100	100	100	100
	%Total Correct Predictions in CV	%Total Correct Predictions	% Total Correct Predictions in CV	% Total Correct Predictions
	100	100	98.87	98.89

## Supplementary Materials

The following are available online at https://www.mdpi.com/2304-8158/10/1/183/s1, Table S1: Data samples (training set and test set). Table S2: Classification results. Figure S1: PCA diagnostic and plots data matrix A_204,18_. Figure S2: PCA diagnostic and plots data matrix C_140,18_. Figure S3: PCA diagnostic and plots data matrix D_70,14_. Figure S4: PCA diagnostic and plots data matrices H_37,421_ and I_28,418_.
