# Supplementary material for: Potentialities of Rapid Analytical Strategies for the Identification of the Botanical Species of Several “Specialty” or “Gourmet” Oils
Source: Foods. 2021 Jan 18;10(1):183. doi: 10.3390/foods10010183 (PMC7831336; doi:10.3390/foods10010183)
Supplement: Supplementary file 1 [file foods-10-00183-s001.zip › supp/Figure S1.docx]

E_TRAINING_SET

SCALING: MEAN CENTERING OR COLUMN CENTERING

PCA


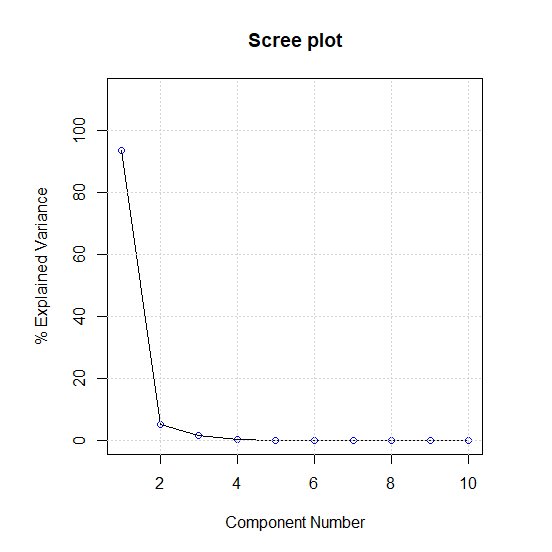


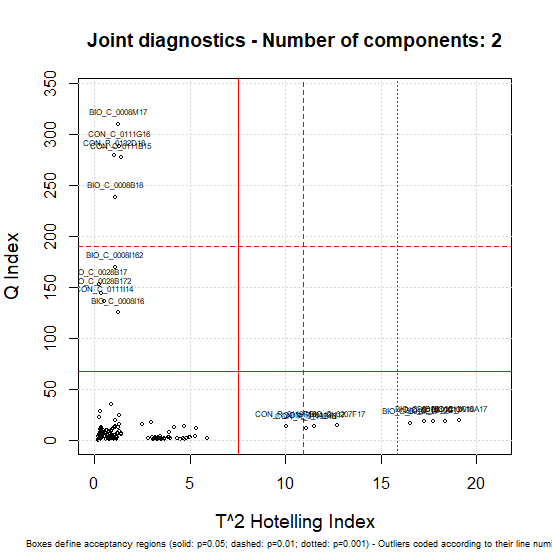


SCALING: AUTOSCALING


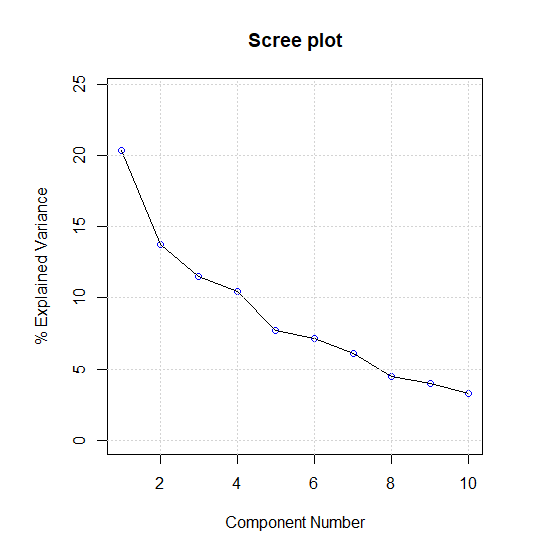


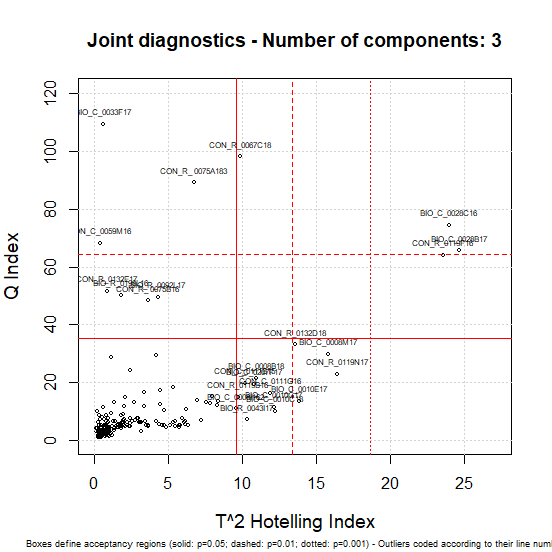


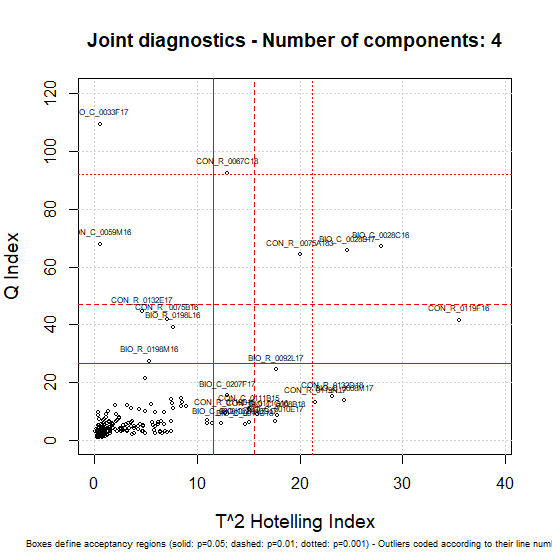


**Figure 1.** PCA diagnostic and plots data matrix A_204,18._
