# Supplementary material for: Potentialities of Rapid Analytical Strategies for the Identification of the Botanical Species of Several “Specialty” or “Gourmet” Oils
Source: Foods. 2021 Jan 18;10(1):183. doi: 10.3390/foods10010183 (PMC7831336; doi:10.3390/foods10010183)
Supplement: Supplementary file 1 [file foods-10-00183-s001.zip › supp/Figure S3.docx]

SOFTWARE CAT

File Name: E2CAT

70 samples (objects)

Apricot 25 samples (objects)

Almond 45 samples (objects)

14 variables

Scaling (Mean Centering or Column Centering)


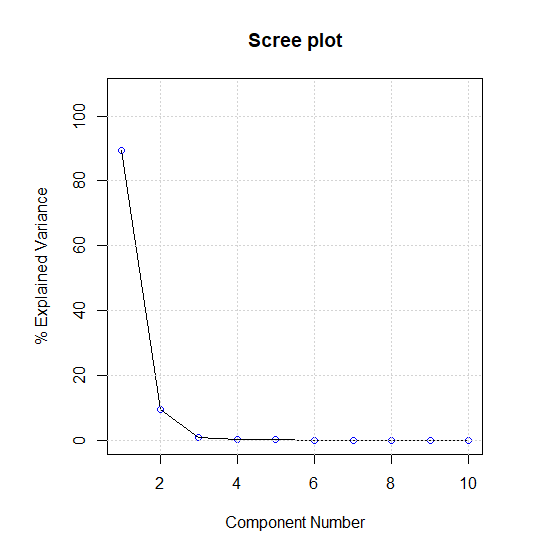


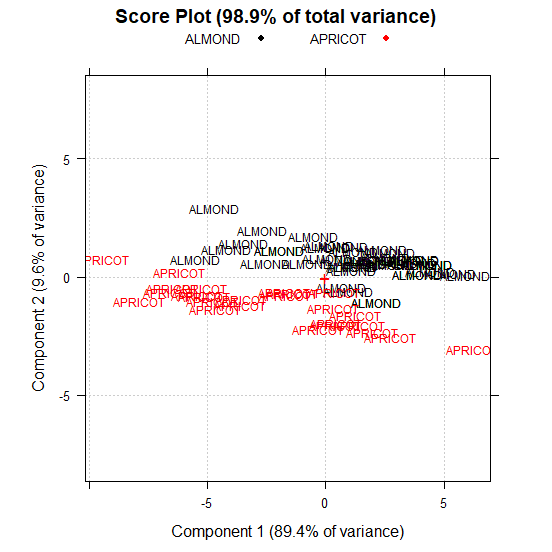


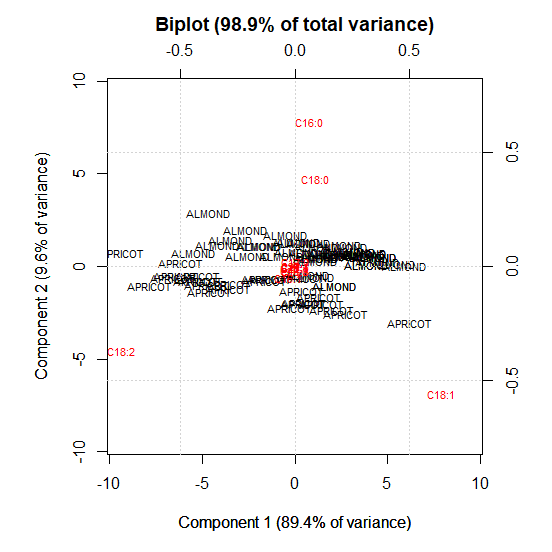

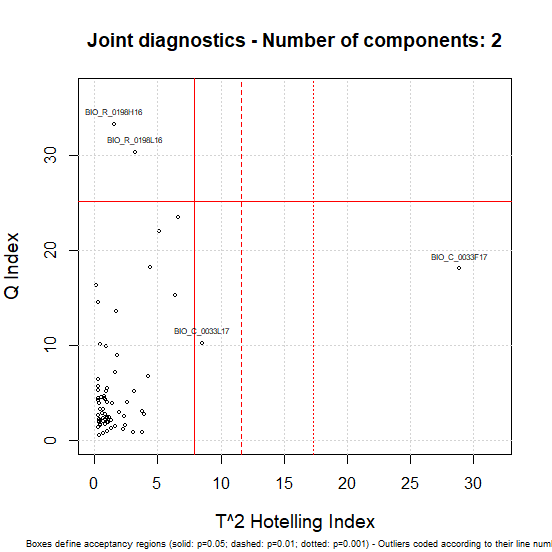


SCALING: AUTOSCALING


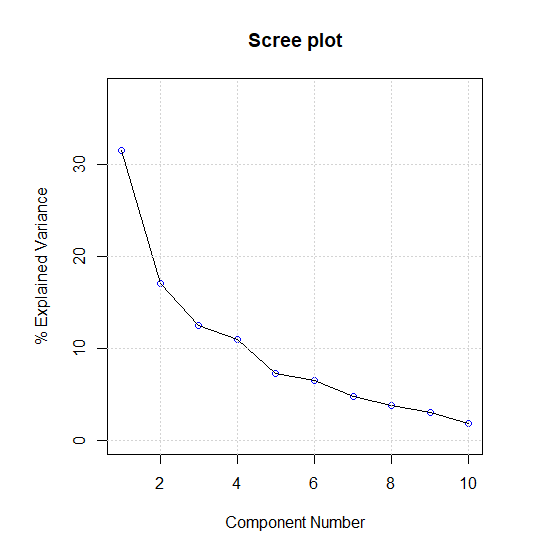


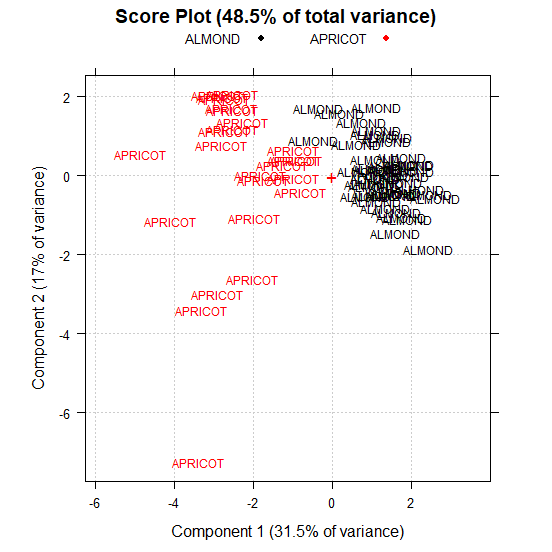


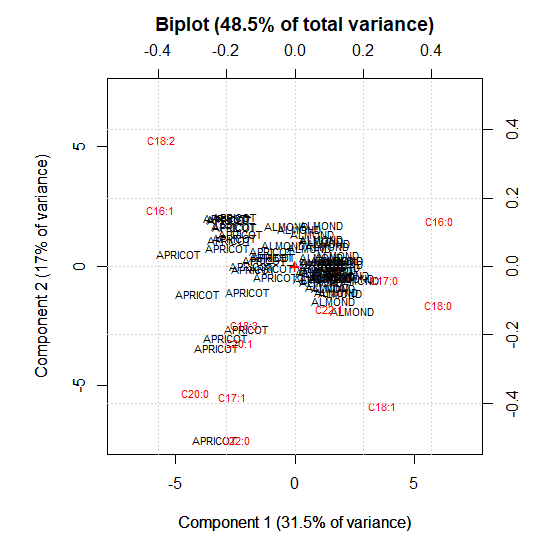

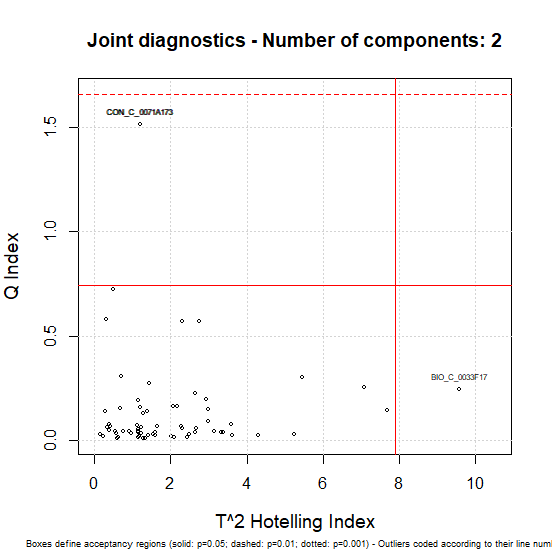


**Figure 3.** PCA diagnostic and plots data matrix D_70,14._
