# Supplementary material for: Potentialities of Rapid Analytical Strategies for the Identification of the Botanical Species of Several “Specialty” or “Gourmet” Oils
Source: Foods. 2021 Jan 18;10(1):183. doi: 10.3390/foods10010183 (PMC7831336; doi:10.3390/foods10010183)
Supplement: Supplementary file 1 [file foods-10-00183-s001.zip › supp/Table S2.docx]

**KNN CV RESULTS (internal prediction results)**

Preprocessing: Autoscale

Cross validation: venetian blinds w/ 5 splits and blind thickness = 1

|  |  | ALMOND | APRICOT | AVOCADO | HAZELNUT | MOSQ | ROS OLIVE | ROSEHIP | SUNFL_HO | SUNFL_LO | WALNUT |
| --- | --- | --- | --- | --- | --- | --- | --- | --- | --- | --- | --- |
| Sensitivity | (Cal): | 1 | 1 | 1 | 1 | 1 | 1 | 1 | 1 | 1 | 1 |
| Specificity | (Cal): | 1 | 1 | 1 | 1 | 1 | 1 | 1 | 1 | 1 | 1 |
| Sensitivity | (CV): | 0.956 | 0.88 | 0.889 | 0.915 | 0.75 | 0.923 | 0.4 | 0.9 | 1 | 1 |
| Specificity | (CV): | 0.962 | 0.978 | 1 | 0.987 | 0.985 | 1 | 0.99 | 1 | 1 | 1 |
| Sensitivity | (Pred): | 1 | 1 | 1 | 1 | 1 | 1 | 1 | 1 | 1 | 1 |
| Specificity | (Pred): | 1 | 1 | 1 | 1 | 1 | 1 | 1 | 1 | 1 | 1 |
| Class. | Err(Cal): | 0 | 0 | 0 | 0 | 0 | 0 | 0 | 0 | 0 | 0 |
| Class. | Err(CV): | 0.0410901 | 0.0711732 | 0.0555556 | 0.0489226 | 0.132653 | 0.0384615 | 0.305025 | 0.05 | 0 | 0 |
| Class. | Err(Pred): | 0 | 0 | 0 | 0 | 0 | 0 | 0 | 0 | 0 | 0 |

Class Err. = average of false positive rate and false negative rate for class,

= 1 - (sensitivity+specificity)/2.

Confusion Matrix (CV):

| **Class:** | **TPR** | **FPR** | **TNR** | **FNR** | **N** | **Err** | **P** | **F1** |
| --- | --- | --- | --- | --- | --- | --- | --- | --- |
| **ALMOND** | 0.911110 | 0.037740 | 0.962260 | 0.088890 | 45 | 0.049020 | 0.872340 | 0.891300 |
| **APRICOT** | 0.880000 | 0.022350 | 0.977650 | 0.120000 | 25 | 0.034310 | 0.846150 | 0.862750 |
| **AVOCADO** | 0.888890 | 0.000000 | 1.000000 | 0.111110 | 9 | 0.004900 | 1.000000 | 0.941180 |
| **HAZELNUT** | 0.936170 | 0.019110 | 0.980890 | 0.063830 | 47 | 0.029410 | 0.936170 | 0.936170 |
| **MOSQ ROSE** | 0.875000 | 0.015310 | 0.984690 | 0.125000 | 8 | 0.019610 | 0.700000 | 0.777780 |
| **OLIVE** | 0.923080 | 0.000000 | 1.000000 | 0.076920 | 13 | 0.004900 | 1.000000 | 0.960000 |
| **ROSEHIP** | 0.200000 | 0.005030 | 0.994970 | 0.800000 | 5 | 0.024510 | 0.500000 | 0.285710 |
| **SUNFLOWER_HO** | 0.900000 | 0.000000 | 1.000000 | 0.100000 | 10 | 0.004900 | 1.000000 | 0.947370 |
| **SUNFLOWER_LO** | 1.000000 | 0.000000 | 1.000000 | 0.000000 | 19 | 0.000000 | 1.000000 | 1.000000 |
| **WALNUT** | 1.000000 | 0.005520 | 0.994480 | 0.000000 | 23 | 0.004900 | 0.958330 | 0.978700 |

Confusion Table (CV):

Actual Class

|  |  |  | **ALMOND** | **APRICOT** | **AVOCADO** | **HAZELNUT** | **MOSQ ROSE** | **OLIVE** | **ROSEHIP** | **SUNFL_HO** | **SUNFL_LO** | **WALNUT** |
| --- | --- | --- | --- | --- | --- | --- | --- | --- | --- | --- | --- | --- |
| **Predicted** | **as** | **ALMOND** | 41 | 3 | 0 | 3 | 0 | 0 | 0 | 0 | 0 | 0 |
| **Predicted** | **as** | **APRICOT** | 4 | 22 | 0 | 0 | 0 | 0 | 0 | 0 | 0 | 0 |
| **Predicted** | **as** | **AVOCADO** | 0 | 0 | 8 | 0 | 0 | 0 | 0 | 0 | 0 | 0 |
| **Predicted** | **as** | **HAZELNUT** | 0 | 0 | 1 | 44 | 0 | 1 | 0 | 1 | 0 | 0 |
| **Predicted** | **as** | **MOSQ ROSE** | 0 | 0 | 0 | 0 | 7 | 0 | 3 | 0 | 0 | 0 |
| **Predicted** | **as** | **OLIVE** | 0 | 0 | 0 | 0 | 0 | 12 | 0 | 0 | 0 | 0 |
| **Predicted** | **as** | **ROSEHIP** | 0 | 0 | 0 | 0 | 1 | 0 | 1 | 0 | 0 | 0 |
| **Predicted** | **as** | **SUNFLOWER_HO** | 0 | 0 | 0 | 0 | 0 | 0 | 0 | 9 | 0 | 0 |
| **Predicted** | **as** | **SUNFLOWER_LO** | 0 | 0 | 0 | 0 | 0 | 0 | 0 | 0 | 19 | 0 |
| **Predicted** | **as** | **WALNUT** | 0 | 0 | 0 | 0 | 0 | 0 | 1 | 0 | 0 | 23 |
| **Predicted** | **as** | **Unassigned** | 0 | 0 | 0 | 0 | 0 | 0 | 0 | 0 | 0 | 0 |

**PREDICTION RESULTS (External Validation)**

| Confusion Matrix: | |  |  |  |  |  |  |  |
| --- | --- | --- | --- | --- | --- | --- | --- | --- |
| Class: | TPR | FPR | TNR | FNR | N | Err | P | F1 |
| **ALMOND** | 1 | 0 | 1 | 0 | 3 | 0 | 1 | 1 |
| **APRICOT** | 1 | 0.1 | 0.9 | 0 | 2 | 0.09091 | 0.5 | 0.66667 |
| **AVOCADO** | 0 | 0 | 1 | 1 | 2 | 0.09091 | NaN | NaN |
| **HAZELNUT** | 1 | 0.05556 | 0.94444 | 0 | 4 | 0.04545 | 0.8 | 0.88889 |
| **MOSQ ROSE** | 1 | 0.04762 | 0.95238 | 0 | 1 | 0.04545 | 0.5 | 0.66667 |
| **OLIVE** | 0.5 | 0 | 1 | 0.5 | 2 | 0.04545 | 1 | 0.66667 |
| **ROSEHIP** | 0 | 0 | 1 | 1 | 1 | 0.04545 | NaN | NaN |
| **SUNFLOWER_HO** | 1 | 0 | 1 | 0 | 2 | 0 | 1 | 1 |
| **SUNFLOWER_LO** | 1 | 0 | 1 | 0 | 2 | 0 | 1 | 1 |
| **WALNUT** | 1 | 0 | 1 | 0 | 3 | 0 | 1 | 1 |

Confusion Table:

Actual Class

|  |  |  | **ALMOND** | **APRICOT** | **AVOCADO** | **HAZELNUT** | **MOSQ ROSE** | **OLIVE** | **ROSEHIP** | **SUNFL_OH** | **SUNFL_LO** | **WALNUT** |
| --- | --- | --- | --- | --- | --- | --- | --- | --- | --- | --- | --- | --- |
| **Predicted** | **as** | **ALMOND** | 3 | 0 | 0 | 0 | 0 | 0 | 0 | 0 | 0 | 0 |
| **Predicted** | **as** | **APRICOT** | 0 | 2 | 2 | 0 | 0 | 0 | 0 | 0 | 0 | 0 |
| **Predicted** | **as** | **AVOCADO** | 0 | 0 | 0 | 0 | 0 | 0 | 0 | 0 | 0 | 0 |
| **Predicted** | **as** | **HAZELNUT** | 0 | 0 | 0 | 4 | 0 | 1 | 0 | 0 | 0 | 0 |
| **Predicted** | **as** | **MOSQ ROSE** | 0 | 0 | 0 | 0 | 1 | 0 | 1 | 0 | 0 | 0 |
| **Predicted** | **as** | **OLIVE** | 0 | 0 | 0 | 0 | 0 | 1 | 0 | 0 | 0 | 0 |
| **Predicted** | **as** | **ROSEHIP** | 0 | 0 | 0 | 0 | 0 | 0 | 0 | 0 | 0 | 0 |
| **Predicted** | **as** | **SUNFLOWER_HO** | 0 | 0 | 0 | 0 | 0 | 0 | 0 | 2 | 0 | 0 |
| **Predicted** | **as** | **SUNFLOWER_LO** | 0 | 0 | 0 | 0 | 0 | 0 | 0 | 0 | 2 | 0 |
| **Predicted** | **as** | **WALNUT** | 0 | 0 | 0 | 0 | 0 | 0 | 0 | 0 | 0 | 3 |
| **Predicted** | **as** | **Unassigned** | 0 | 0 | 0 | 0 | 0 | 0 | 0 | 0 | 0 | 0 |

**LDA RESULTS (SOFTWARE CAT)**

SCALING: AUTOSCALING

Confusion Matrix in Cross Validation

**ALMOND APRICOT**

ALMOND 45 0

APRICOT 0 25

[1] % Correct Predictions in Cross Validation

ALMOND APRICOT

100 100

[1] % Total Correct Predictions in Cross Validation

[1] 100

**KNN RESULTS (SOFTWARE PLS-TOOLBOX)**

Preprocessing: Autoscale

Cross validation: venetian blinds w/ 5 splits and blind thickness = 1

Statistics for each y-block column:

Sensitivity (Cal): 1.000 1.000

Specificity (Cal): 1.000 1.000

Sensitivity (CV): 0.956 0.920

Specificity (CV): 0.920 0.956

Sensitivity (Pred): 1.000 1.000

Specificity (Pred): 1.000 1.000

Class. Err (Cal): 0 0

Class. Err (CV): 0.0622222 0.0622222

Class. Err (Pred): 0 0

Class Err. = average of false positive rate and false negative rate for class,

= 1 - (sensitivity+specificity)/2.

**CV RESULTS (Internal Prediction)**

Confusion Matrix (CV):

Class: TPR FPR TNR FNR N Err P F1

ALMOND 0.95556 0.00000 1.00000 0.04444 45 0.02857 1.00000 0.97727

APRICOT 1.00000 0.04444 0.95556 0.00000 25 0.02857 0.92593 0.96154

Matthew's Correlation Coefficient = 0.941

Confusion Table (CV):

Actual Class

ALMOND APRICOT

Predicted as ALMOND 43 0

Predicted as APRICOT 2 25

Predicted as Unassigned 0 0

**PREDICTION RESULTS (External Prediction)**

Confusion Matrix:

Class: TPR FPR TNR FNR N Err P F1

ALMOND 1.00000 0.00000 1.00000 0.00000 3 0.00000 1.00000 1.00000

APRICOT 1.00000 0.00000 1.00000 0.00000 2 0.00000 1.00000 1.00000

Matthew's Correlation Coefficient = 1.000

Confusion Table:

Actual Class

ALMOND APRICOT

Predicted as ALMOND 3 0

Predicted as APRICOT 0 2

Predicted as Unassigned 0 0
