# Supplementary figures and images for: Potentialities of Rapid Analytical Strategies for the Identification of the Botanical Species of Several “Specialty” or “Gourmet” Oils
Source: Foods. 2021 Jan 18;10(1):183. doi: 10.3390/foods10010183 (PMC7831336; doi:10.3390/foods10010183)

FUSI


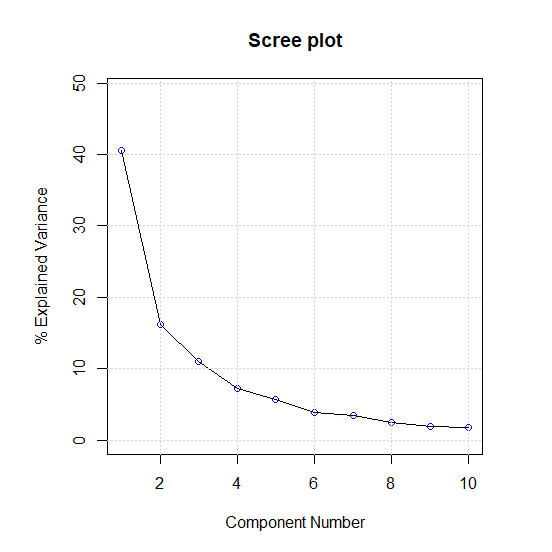


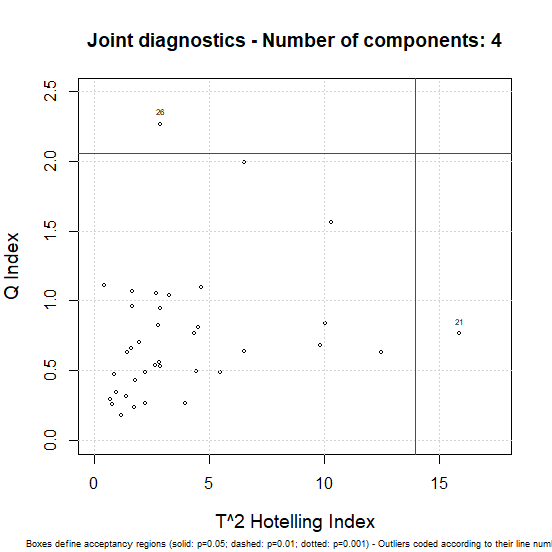


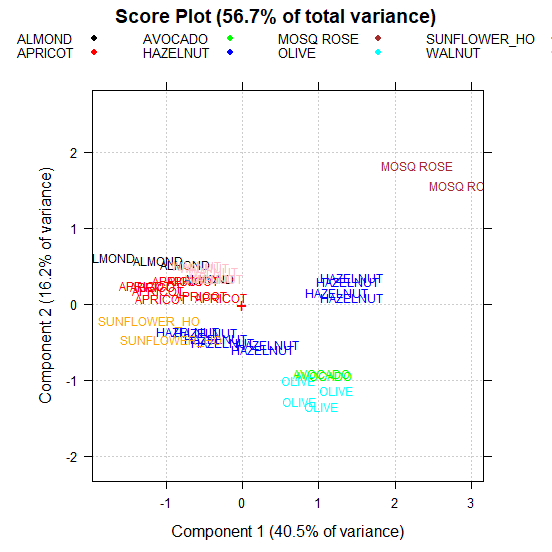


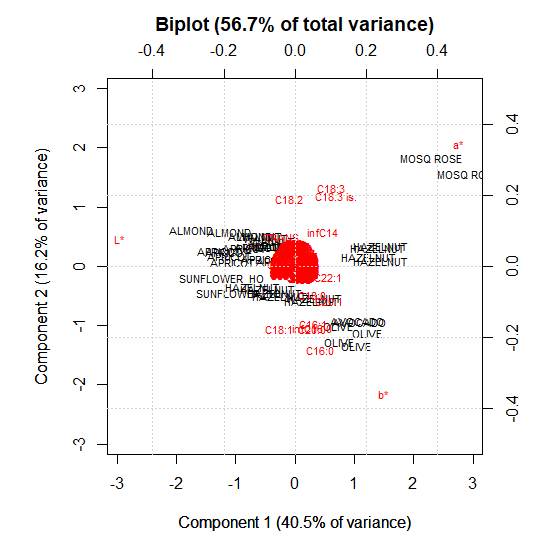


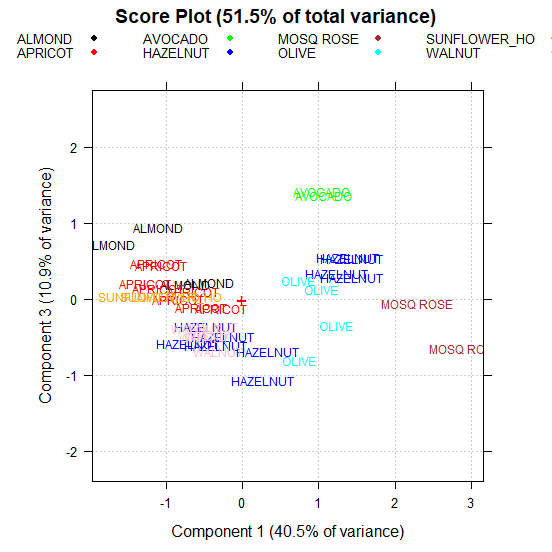


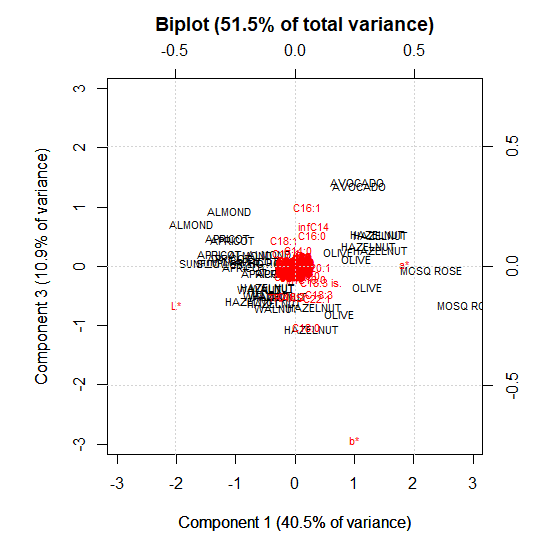


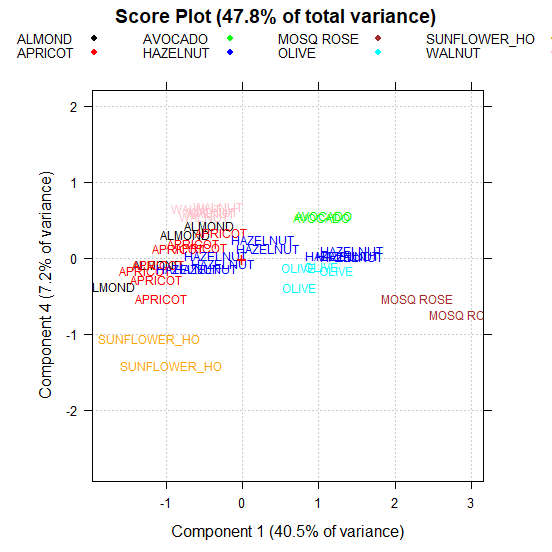


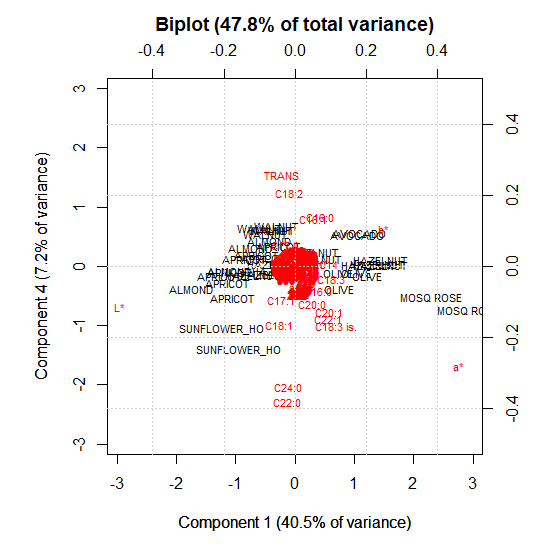


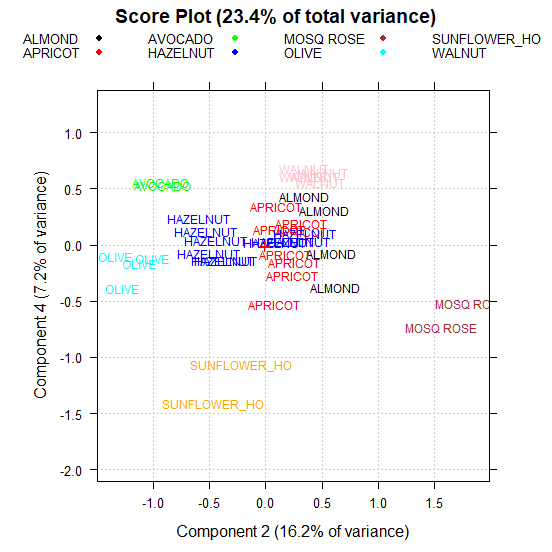


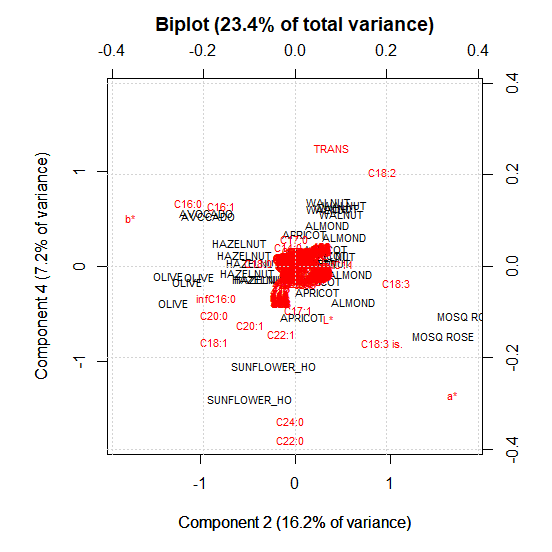


FUSI5CAT.blsc


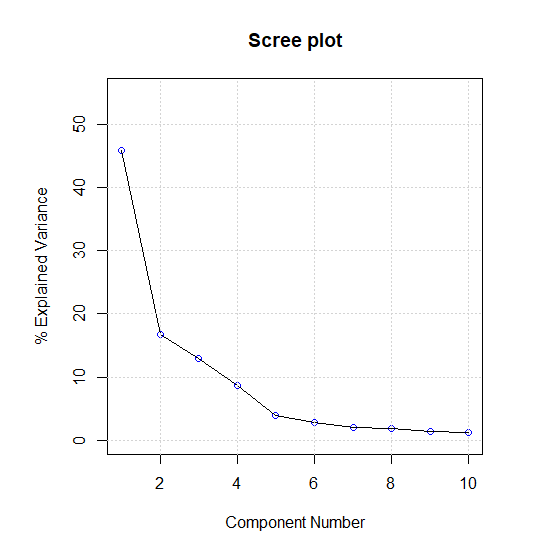


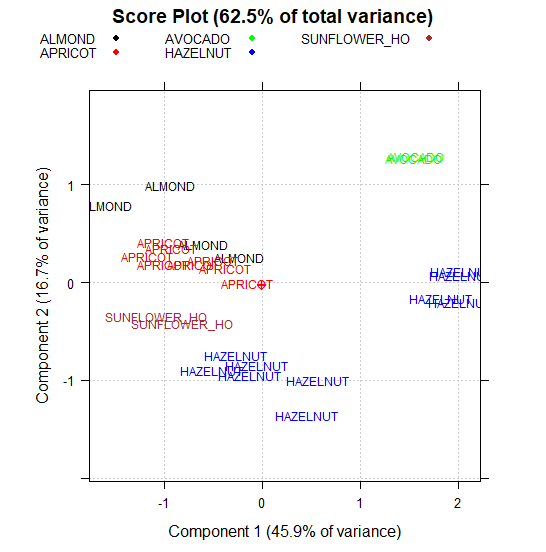


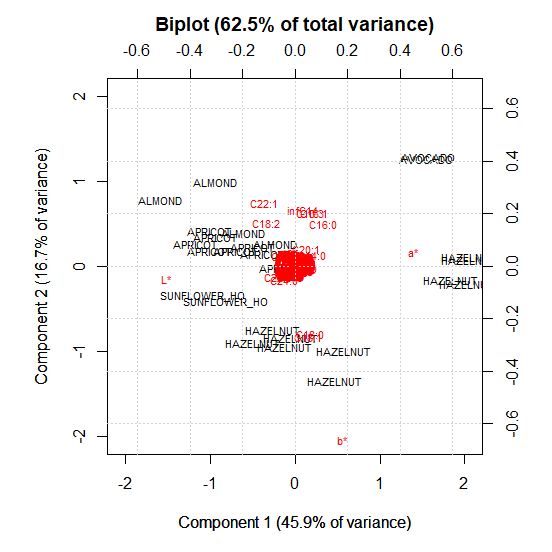


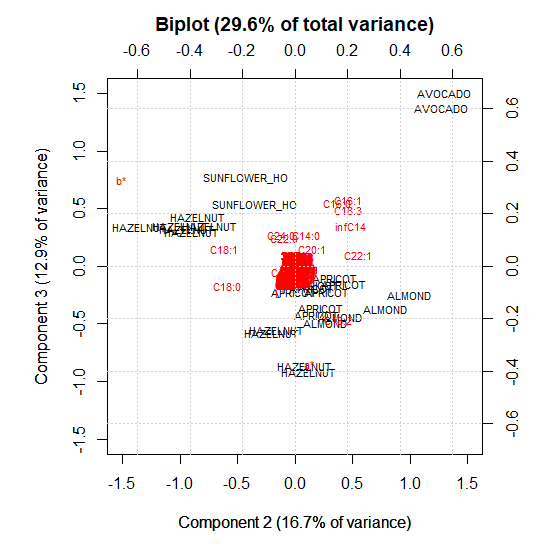


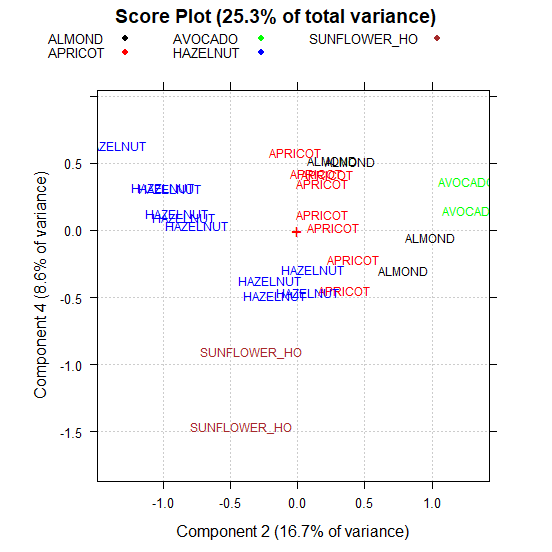


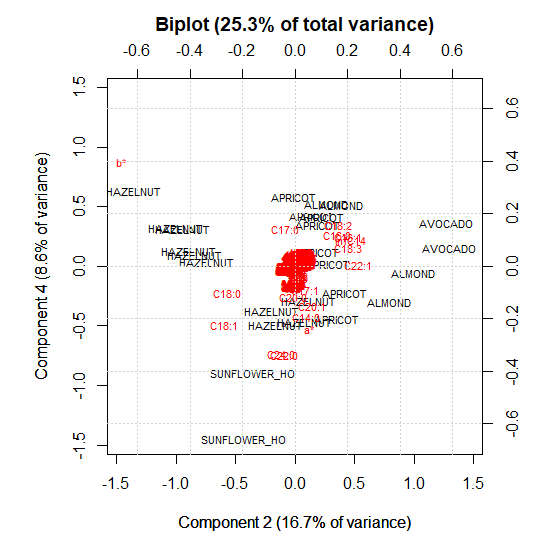


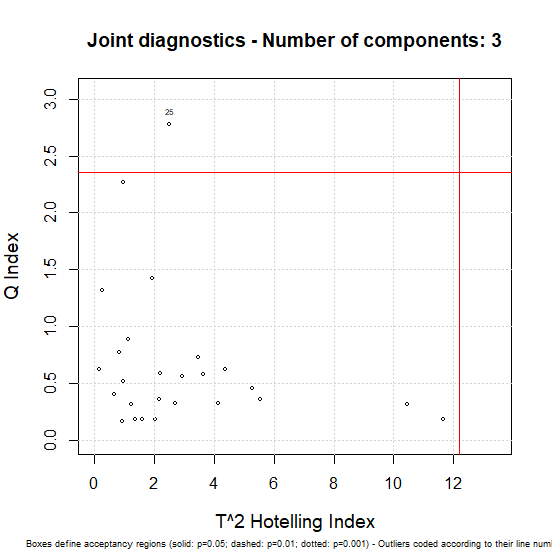


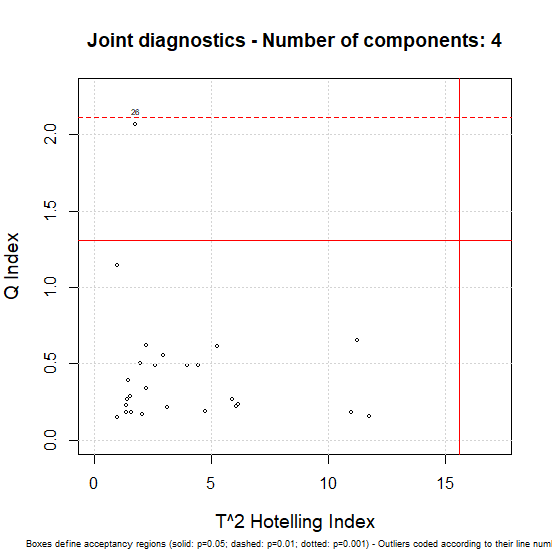


**Figure 4.** PCA diagnostic and plots data matrices H37,421 andI28,418.

Supplement: Supplementary file 1 [file foods-10-00183-s001.zip › supp/Figure S4.docx]
